# Musculoskeletal Pathology in Cerebral Palsy: A Classification System and Reliability Study

**DOI:** 10.3390/children8030252

**Published:** 2021-03-23

**Authors:** H. Kerr Graham, Pam Thomason, Kate Willoughby, Tandy Hastings-Ison, Renee Van Stralen, Benan Dala-Ali, Peter Wong, Erich Rutz

**Affiliations:** 1 Department of Paediatrics, University of Melbourne, Parkville, VIC 3052, Australia; erich.rutz@rch.org.au; 2 Hugh Williamson Gait Laboratory, Royal Children’s Hospital, Parkville, VIC 3052, Australia; pam.thomason@rch.org.au (P.T.); tandy.hastingsison@rch.org.au (T.H.-I.); peter@wongs.net.au (P.W.); 3 Murdoch Children’s Research Institute, Parkville, VIC 3052, Australia; kate.willoughby@rch.org.au; 4 Orthopaedic Department, The Royal Children’s Hospital, Parkville, VIC 3052, Australia; 5 Department of Orthopedic Surgery, Sophia Children’s Hospital, Erasmus MC, 3015GD Rotterdam, The Netherlands; r.a.vanstralen@gmail.com; 6 Orthopaedic Department, Great Ormond Street Hospital for Children, Great Ormond Street, London WC1N 3JH, UK; benan@doctors.org.uk

**Keywords:** cerebral palsy, musculoskeletal pathology, spasticity, contracture, deformity, decompensation

## Abstract

This article presents a classification of lower limb musculoskeletal pathology (MSP) for ambulant children with cerebral palsy (CP) to identify key features from infancy to adulthood. The classification aims to improve communication, and to guide referral for interventions, which if timed appropriately, may optimise long-term musculoskeletal health and function. Consensus was achieved by discussion between staff in a Motion Analysis Laboratory (MAL). A four-stage classification system was developed: Stage 1: Hypertonia: Abnormal postures are dynamic. Stage 2: Contracture: Fixed shortening of one or more muscle-tendon units. Stage 3: Bone and joint deformity: Torsional deformities and/or joint instability (e.g., hip displacement or pes valgus), usually accompanied by contractures. Stage 4: Decompensation: Severe pathology where restoration of optimal joint and muscle-tendon function is not possible. Reliability of the classification was tested using the presentation of 16 clinical cases to a group of experienced observers, on two occasions, two weeks apart. Reliability was found to be very good to excellent, with mean Fleiss’ kappa ranging from 0.72 to 0.84. Four-stages are proposed to classify lower limb MSP in children with CP. The classification was reliable in a group of clinicians who work together. We emphasise the features of decompensated MSP in the lower limb, which may not always benefit from reconstructive surgery and which can be avoided by timely intervention.

## 1. Introduction

Cerebral palsy (CP) is the most common cause of life-long physical disability in most developed countries, with a prevalence ranging from 1.5 to 3 per 1000 live births [1]. The accepted international definition of CP is:

“*Cerebral palsy describes a group of permanent disorders of the development of movement and posture, causing activity limitation, that are attributed to non-progressive disturbances that occurred in the developing fetal or infant brain. The motor disorders of cerebral palsy are often accompanied by disturbances of sensation, perception, cognition, communication and behaviour, by epilepsy and by secondary musculoskeletal problems.*”

[1]

This definition highlights the secondary musculoskeletal pathology (MSP), which the majority of children will develop with time. The MSP may affect many aspects of the child’s function, limiting their physical activity, participation and quality of life. Many studies have reported the progressive nature of MSP [2,3].

In 1990, Dr Mercer Rang proposed a scheme to describe the musculoskeletal stages in the life of a child with spastic cerebral palsy (CP) [2]. Stage I was characterised by spasticity, Stage II by contractures and Stage III by bony deformities. Whilst this was the first outline and useful teaching aid, it did not encapsulate the complexities of musculoskeletal pathology (MSP) nor provide a complete template for communication, education and management. In 2010, Dr Jon Davids classified three stages of progressive foot deformity in children with CP and related each stage to treatment options [3].

There is currently no accepted classification for musculoskeletal pathology in children with cerebral palsy, apart from the three group illustration drawn by Dr Mercer Rang [2]. However, his scheme has never really been adopted or used within the literature as a basis for classifying children, as an aid to decision-making regarding management or as a tool for clinical research. The classification that we are proposing in this study is the first of its kind. This makes our work somewhat more difficult in that we do not have a direct comparator. In this first study, we address the classification of MSP and its reliability [1,2,3].

In 2002, Rosenbaum et al., created gross motor curves describing motor development in children with CP. There are five curves that relate to the five levels of the Gross Motor Function Classification System (GMFCS) [4,5]. Gross motor skills are achieved quickly in the first two years of life, before beginning to slow and then plateau between four to six old. Between six and twelve, gross motor function deteriorates at GMFCS Levels III, IV and V, which is coincident with the pubertal growth spurt and the progression of musculoskeletal deformities [4,5]. In 2014, Mudge et al., published normative reference values for lower limb joint range of motion (ROM), bony torsion and alignment in typically developing children (TDC) aged four to sixteen [6]. The key finding of their study was that in TDC, joint ROM decreases over time, with age and onset of skeletal maturity. Nordmark et al., 2009, also reported decreasing ROM in the lower limb joints of children with CP over time [7]. They reported a decreasing mean ROM between the age of two and fourteen in all joints measured. The decrease in joint ROM varied according to GMFCS level and CP subtype [7,8].

In 2008, Hagglund et al., reported that in a population of children with CP aged between 0 to 15 years of age, muscle tone, as measured by the Modified Ashworth Scale, increased up to the age of four, followed by a spontaneous decrease in muscle tone each year up until age twelve [9]. The MAS is a six-level ordinal scale from zero, (no increase in tone) to four (the body part is rigid in flexion or extension). The higher the score, the more spasticity is felt on the passive motion of the joint [1,10].

At age four, 47% of children in the Hagglund study had spasticity in the gastrocsoleus muscle defined as Modified Ashworth Scale (MAS) of II–IV. However, by age twelve, only 23% of the study population had that level of spasticity [9].

In our tertiary children’s hospital MAL, we receive referrals of children with CP from early childhood through to adult life. We assess the suitability of spasticity management, including oral medications, injections of botulinum neurotoxin A (BoNT-A), selective dorsal rhizotomy (SDR) and the use of intrathecal baclofen. We perform a 3-dimensional gait analysis to identify gait deviations, musculoskeletal impairments and make recommendations for multilevel surgery using a diagnostic matrix [10,11,12]. We see a substantial number of children with late MSP for which it is impossible to restore normal structure and function, or where such pathology can only be managed by salvage surgical procedures [1,11]. We also experience a substantial diversity of opinion regarding the age and stage at which different interventions might be most appropriate for a child. A review of late presenting children suggests a lack of clarity regarding both optimal timing for interventions, and an understanding of the stages in the progression of lower limb MSP [1,11].

This study presents a classification system for MSP in children with CP, informed by these “natural history” studies and to test the reliability [5,6,7,8,9].

## 2. Materials and Methods

### 2.1. Development of a Classification System

**Figure 1:**
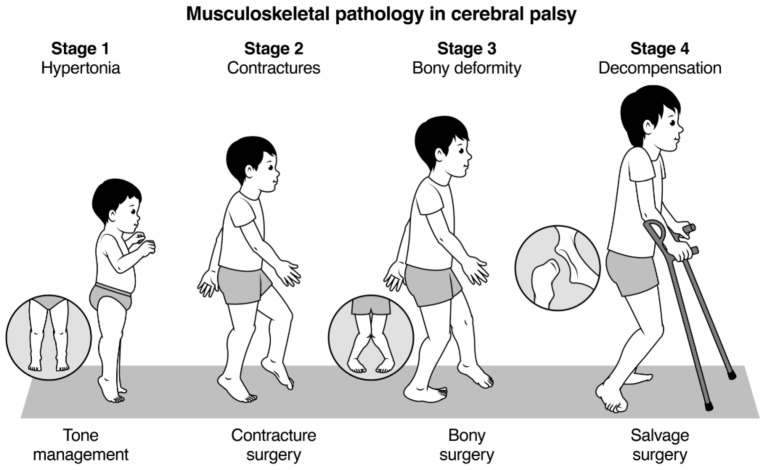
The stages of musculoskeletal pathology (MSP) in children with spastic cerebral palsy, from birth to skeletal maturity. Note the overlapping age ranges, and that features of Stage 2 and 3 usually occur together.

The publications by Rang and Davids were discussed, and principal limitations noted [1,3]. We reviewed referral data for interventions, such as injection of BoNT-A for lower limb spasticity, assessments of children for SDR and for multilevel surgery over a 12-month period. We constructed a classification that would be clinically useful, easy to communicate, and easy to teach. We emphasise that the stages have a relationship to the age of the child and that overlap exists between each stage, with particular emphasis on the overlap that occurs between children with MSP at Stages 2 and 3. The majority of ambulant children with CP have combinations of soft tissue contracture and bony torsion, combined with residual issues related to spasticity and weakness [9,10,11,12,13]. In addition, we wanted to draw attention to the risk of decompensated pathology at each anatomic level in adolescents and young adults with CP [1,11]. A four-stage classification was developed (Figure 1, Table 1).

### 2.2. Stage I: Hypertonia: From Birth to Age 4–6 Years

**Figure 2:**
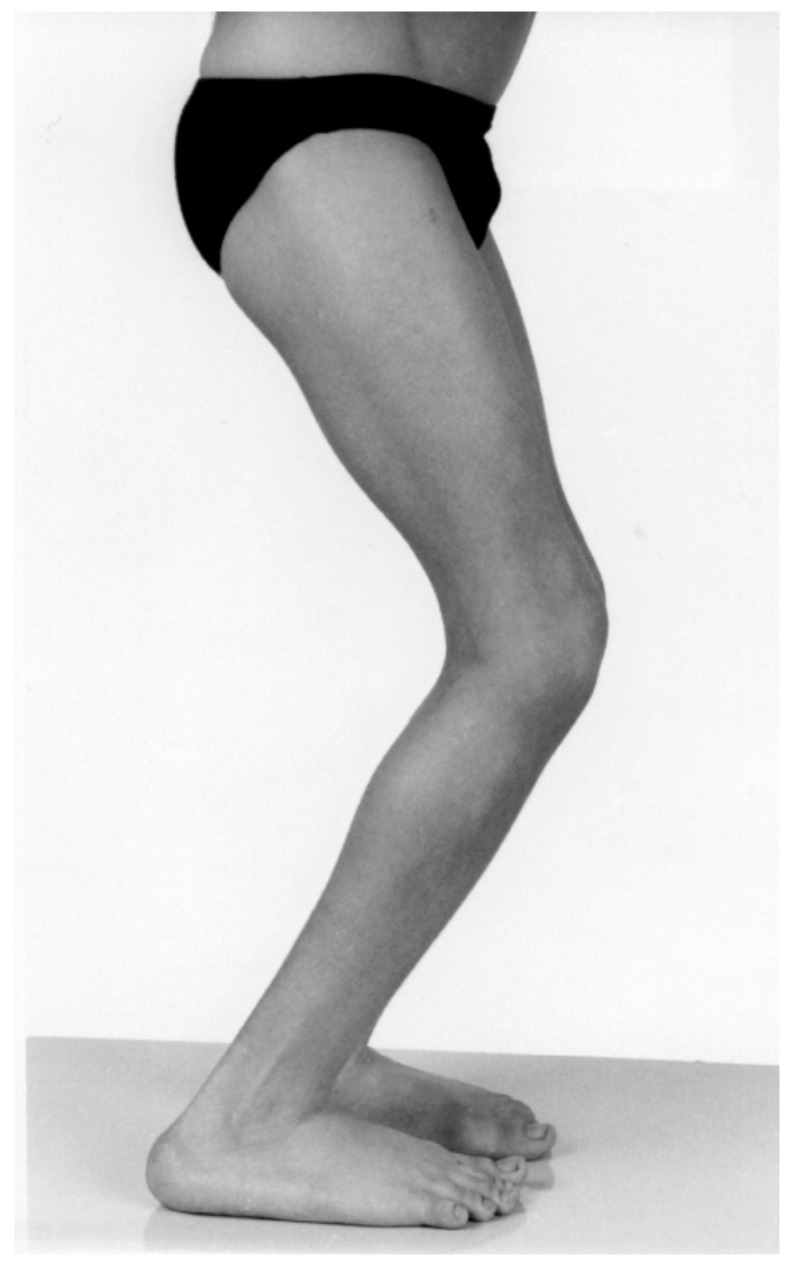
A 10-year old boy with spastic diplegia, GMFCS III with iatrogenic crouch gait after bilateral TALs at age 4. The surgeon recorded in the operation note “minimal fixed contracture but severe toe walking”. Equinus in diplegia at age 4 is usually more spastic than fixed and is more safely managed by injections of BoNT-A and AFOs. The MSP at index surgery was Stage 1 and is now Stage 4. There is no reliable intervention for the overlengthened heel-cord.

Stage 1 is the typical CP clinical phenotype from identification or diagnosis of CP to approximately age four to six. (Figure 1, Table 1) The principal issues relate to hypertonia (spasticity, dystonia and mixed movement disorders) and delayed acquisition of gross motor milestones [1,2,13]. During this stage, children have few if any contractures and orthopaedic surgery is not usually needed, and surgical outcomes may be unpredictable, occasionally disastrous [11,14] (Figure 2). The focus of management is early intervention to promote acquisition of gross motor function, often using a combination of physiotherapy, spasticity management, and the use of ankle foot orthoses and assistive devices. Spasticity management may include injections of BoNT-A for focal spasticity, such as spastic equinus. Typical postures at each anatomic level are described in Table 1. After age 5, spasticity decreases and contracture increases [1,9].

### 2.3. Stage 2: Contractures: Age 4–12

Over time, children with CP develop decreasing joint ROM, related to a progressive mismatch between the length of muscle-tendon units (MTUs) and the neighbouring long bone [1,11]. (Figure 1, Table 1) This is the stage when soft tissue contractures are noted on physical examination and may contribute to the impairment of gait and function. Management of children in Stage 2 is usually by orthopaedic surgery to correct contractures by muscle recessions and various forms of MTU lengthening and tendon transfers [11,15,16,17]. Threshold values for contractures at the major lower limb joints have not been clearly defined. For practical purposes, we have chosen ankle dorsiflexion less than neutral when the knee is extended and any degree of fixed flexion deformity at the knee or hip. These values are substantially lower than the values for TDC [6]. At Stage 2 single level (type II hemiplegia) or multilevel (diplegia) muscle tendon lengthening may be the optimal solution, keeping in mind the effects of spasticity and lower limb weakness [10,11].

### 2.4. Stage 3: Bony Deformity: Age 4–12

**Figure 3:**
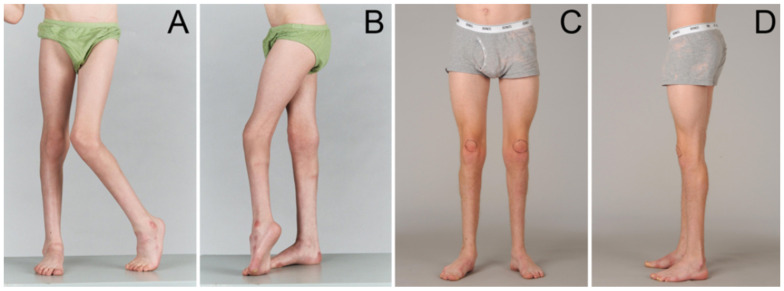
Stage 3 MSP in a 10-year old boy with very asymmetric spastic diplegia, before (**A**,**B**) and five years after SEMLS. (**C**,**D**) All the deformities were corrected with conventional SEMLS procedures. He had a marked improvement in gait and function with no relapse at five-year follow-up. No additional interventions for spasticity or contractures were required.

The majority of children with CP who develop contractures have concomitant evidence of bony deformity. Increased femoral neck anteversion (FNA) is present from birth in the majority of children with CP and is probably not primarily caused by spasticity [11,18]. (Figure 1, Table 1) We define increased FNA as >25 degrees, as this is a threshold value for considering surgical intervention [12,13]. External tibial torsion (ETT), in both typically developing children and in children with CP, appears to develop with time. Joint instability, including hip dysplasia, which is less common in ambulant children and generally milder than in non-ambulant children, may also be present [3,11]. Midfoot instability with a breakdown of the midfoot and pes valgus is a common accompaniment of equinus contracture [3,11]. At Stage 3, rotational osteotomies and joint stabilisation procedures may be appropriate. These are most often conducted in combination with soft tissue surgery as part of multilevel surgery or Single Event Multilevel Surgery (SEMLS) [11,16,17] (Figure 3).

NB: Spasticity reduces with age in many children with CP, and soft tissue surgery may further reduce muscle tone [9,11]. However, a few children may need spasticity management even after successful orthopaedic reconstructive surgery.

### 2.5. Stage 4: Decompensation: Age 10 to Adulthood

**Figure 4:**
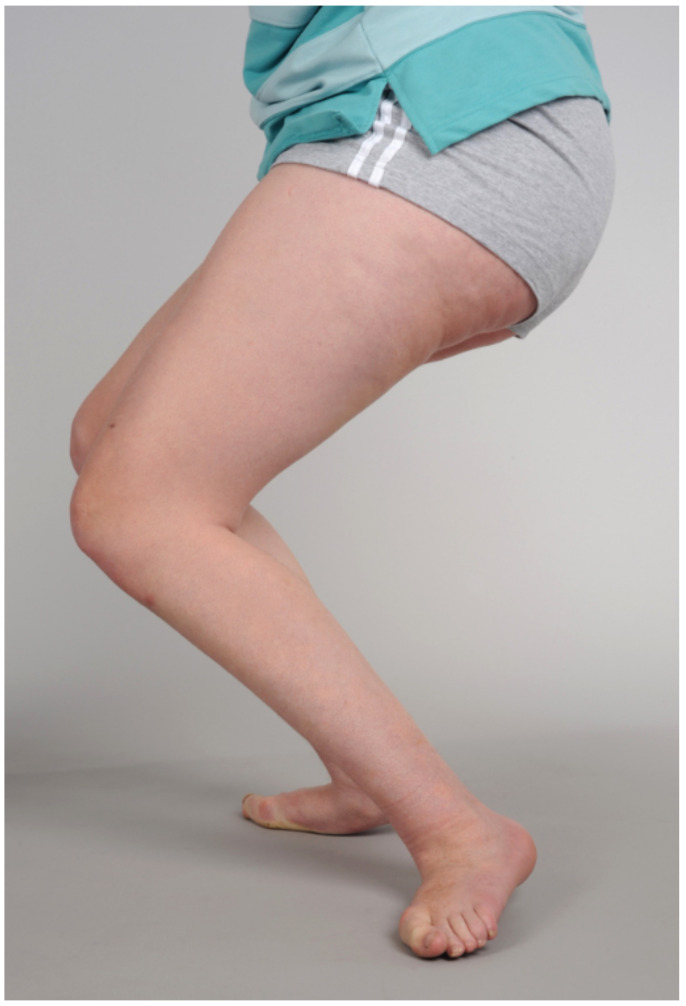
Decompensated pathology with severe crouch gait in a 15-year old girl with spastic diplegia, GMFCS IV (previously GMFCS III) There was no prior intervention apart from injections of BoNT-A. These are “natural history deformities”. The knee flexion contractures measured 45 degrees bilaterally, and the knees were flexed almost 90 degrees during gait. The feet had severe pes valgus and painful hallux valgus. The MAL team concluded that the MSP was Stage 4 with severe decompensation and advised that surgery was unlikely to be beneficial. Bilateral DFEOs and PTS were performed and were accompanied by neurovascular injuries, loss of ambulatory ability and deterioration in transfer ability.

**Figure 5:**
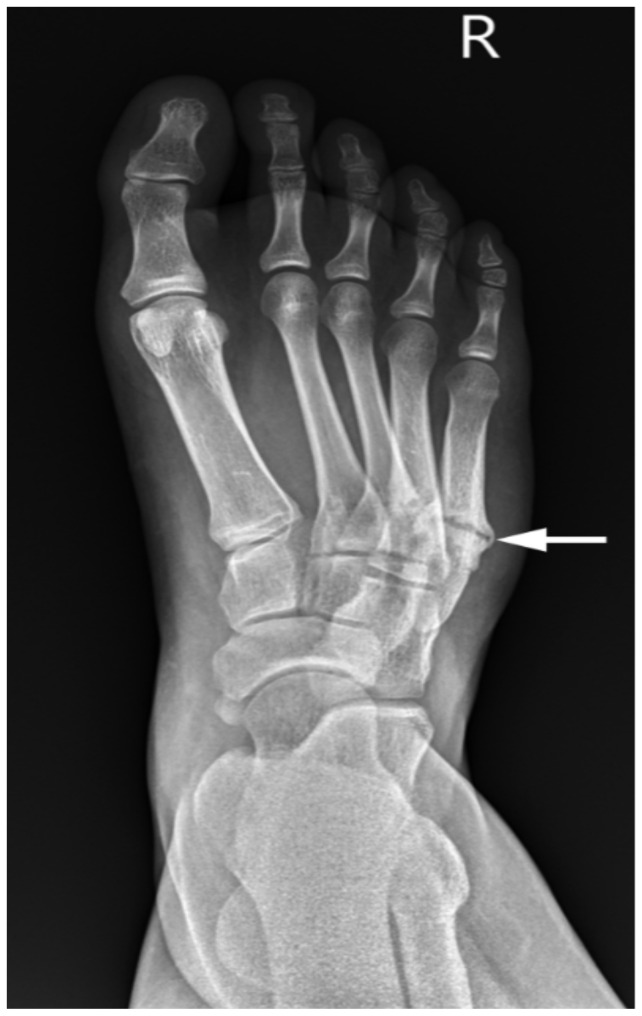
Radiographic features of Stage 4 MSP in the foot. Severe varus deformity of the foot in a 14-year old boy with Type IV hemiplegia, GMFCS II. There are healing fractures of the 4th and 5th metatarsals, from severe chronic overloading. Management to date has been injections of BoNT-A to the gastrocsoleus and tibialis posterior.

**Figure 6:**
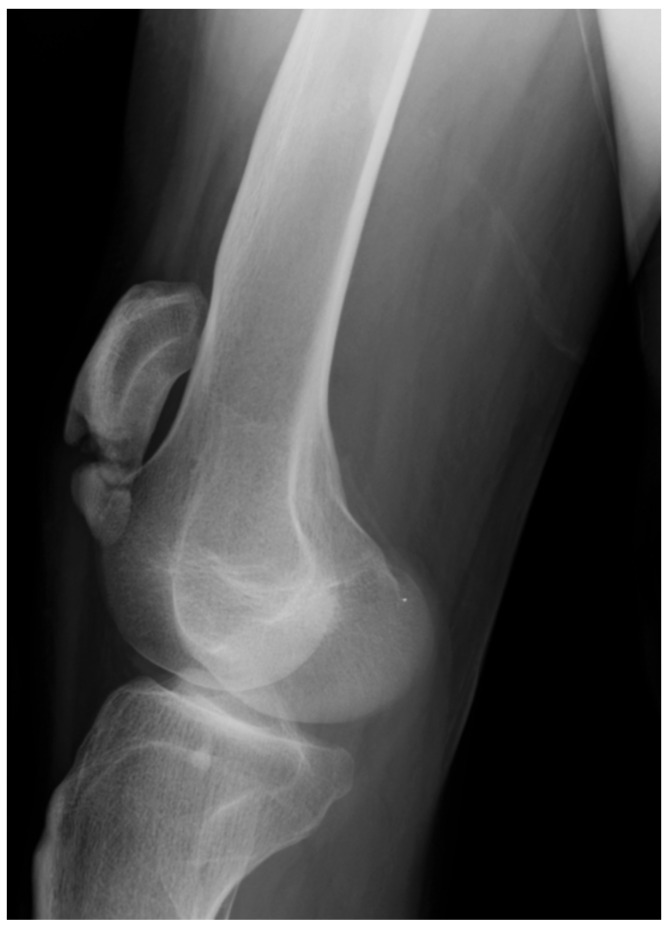
Radiographic features of Stage 4 MSP in the knee, in a 16-year old boy with severe crouch gait. There is marked patella alta and stress fracture of the patella with signs of healing with separation at the fracture site.

**Figure 7:**
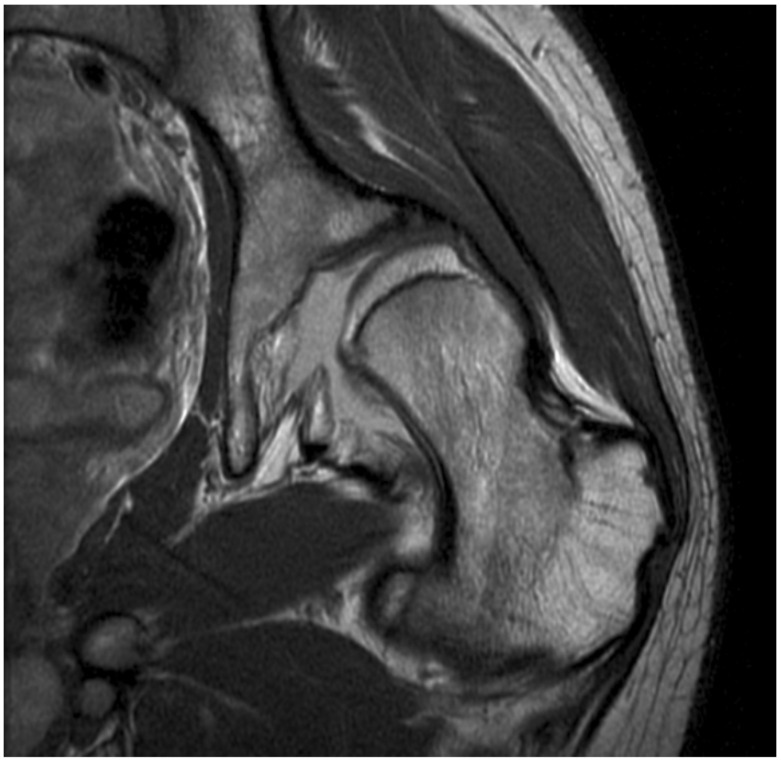
Radiographic features of Stage 4 MSP at the hip: MRI scan of a 14-year old boy, Type IV, left hemiplegia, chronic neglected hip displacement, GMFCS III. Note the full thickness loss of articular cartilage on the lateral aspect of the femoral head. The left hip was successfully reconstructed, but this is salvage surgery. The lost cartilage does not regenerate, and the hip is destined for premature arthrosis and arthroplasty.

Decompensation indicates that the MSP has progressed to a point where restoration of optimal joint and muscle-tendon function is no longer possible. In general, this is more frequently seen after the pubertal growth spurt, but is occasionally seen in younger children [1,11]. (Figure 1 and Figure 4, Table 1) Key features of decompensation include severe joint contractures and bony deformity, as well as contractures of MTUs, weakness and hypertonia (Figure 2, Figure 4, Figure 5, Figure 6 and Figure 7).

The classic area of decompensated MSP is the hip joint when progressive subluxation leads to femoral head deformity and loss of articular cartilage (Figure 7). Even though a hip in this state may be reconstructed, articular cartilage will not regenerate, and the hip cannot be returned to the previous level of mobility and function [19]. Even though there are effective surgical strategies for the correction of severe crouch gait, which include distal femoral extension osteotomy (DFEO) and patella tendon shortening (PTS), these operative strategies are best described as “salvage surgery” rather than “primary reconstructive surgery” [20,21]. Salvage surgery also includes loss of synovial joints, due to arthrosis, requiring arthrodesis or arthroplasty. In younger children with flexible deformities, both equinovarus and pes valgus can be successfully corrected by joint sparing procedures [2]. However, if foot and ankle deformities are neglected, deformities of tarsal bones and joint surfaces may result in fixed deformity and a need for joint excision and arthrodesis, which are also classified as salvage surgery rather than primary reconstructive surgery (Table 1). Prolonged, excessive loading of the lateral border of the varus foot may result in skin callosities and breakdown and stress fractures of the 4th and 5th metatarsals [11] (Figure 5).

### 2.6. Reliability Testing

In order to evaluate the reliability of the MSP classification system, three orthopaedic surgeons and six physiotherapists working together in a MAL were asked to view and grade MSP in 16 clinical scenarios. (Table 2) All participants had experienced in the assessment and management of individuals with CP from infancy to early adult life. The participants attended a familiarisation session where the MSP classification was explained, and illustrations and written information were supplied. These 16 scenarios were derived from children with a confirmed diagnosis of CP who had previously attended the MAL for assessment of gait and function and to plan intervention. The scenarios were selected to cover the typical age range for the MAL referrals, and all four grades of the classification were included. All children included in the exercise had both pre and post intervention MAL data which confirmed that the intervention selected had been successful, using objective criteria from the diagnostic matrix [10,11]. Each scenario consisted of a summary of clinical history, classification by gross motor function, physical examination data, relevant radiographic findings and a video recording of gait within standard gait laboratory conditions [10]. Each participant was asked to grade the MSP in each scenario according to the MSP classification system. The classification was repeated two weeks later with the order of presentation of the clinical scenarios changed. Unweighted Fleiss’ κ statistics with 95% confidence intervals (CI) were used to determine inter-rater reliability and intra-rater reliability. A κ score of 0 to 0.2 was deemed as poor, 0.21 to 0.4 as fair, 0.41 to 0.6 as good, 0.61 to 0.8 as very good and 0.81 to 1.0 as excellent [22]. Calculations were performed in Stata version 14.3 (StataCorp, College Station, TX, USA).

## 3. Results

The mean age of children included in the clinical scenarios was 8 + 6 years (range from 1 + 8 to 15 + 9 years). Three were GMFCS I, nine were GMFCS II, and four were GMFCS III at the time of assessment. Three were MSP Stage 1, three were MSP Stage 2, five were MSP Stage 3, and five were MSP Stage 4, as graded by the senior author. Inter-rater reliability was very good on the first reading (κ, 0.78; CI 0.72–0.85) and improved on the second reading (κ, 0.82; CI 0.75–0.89) Intra-rater reliability was excellent (κ, 0.84; CI 0.80–0.86). There were no significant differences in reliability between surgeons and physiotherapists, or across the four MSP grades.

## 4. Discussion

Gross motor function in children with CP exists as a continuum when measured by GMFM, but classification by GMFCS into just five groups has proven to be a valid, reliable and practical method to describe the prognosis and goal setting across the full spectrum function of children with CP [4,5,23]. We propose a simple four-stage classification of lower limb MSP in children with CP, recognising that this too is a simplification of multiple, complex and nuanced clinical phenotypes (Figure 1, Table 1 and Table 2). Just as GMFCS is a useful tool to subdivide the gross motor function of the CP population into five phenotypic groups, each with its own long-term prognosis and functional implications, we think that a classification of lower limb MSP, which is also on a continuous spectrum, might be useful for education, communication and research purposes [23].

Broad classifications in CP may help clinicians understand the prognosis and avoid category errors in management. For example, the management of children at GMFCS IV is challenging. As with all children with CP, there is early acquisition of gross motor function, which may lift hopes that the child will become a functional ambulator [4,5,23]. However, in early childhood, the gross motor curve plateaus and then declines, and after puberty, functional ambulation is lost. In our view, it would be a “category error” to offer SEMLS to a child who functions at GMFCS IV with the aim of improving gait function [11,21,23].

Before the availability of botulinum toxin, many children with CP had early, single-level surgery for equinus gait, even when the problem was more spastic than fixed contracture. The outcomes have been reported to be disastrous in several studies [11,14] (Figure 2). Now the pendulum may have swung too far the other way, and some adolescents are treated with injections of BoNT-A at MSP Stages 2, 3 and 4 (Figure 4 and Figure 5). Some children may be offered SDR when the number and severity of contractures and lever arm deformities preclude a good outcome. These are examples of category errors in staging MSP.

In children with Stage 1 MSP, equinus should not be managed by orthopaedic surgery, but by methods that manage hypertonia, preserve muscle tendon function and promote improvements in gross motor function and gait [11,14] (Figure 2). Equally, spasticity measures are much less effective at Stages 2 and 3, and orthopaedic surgery is the intervention of choice (Figure 3).

Given the long-term sequelae of pain, stiffness and arthrosis, which accompany decompensated MSP, we draw attention to the stage of decompensation at each anatomic level to encourage regular examination, assessment, timely referral and intervention to avoid irreparable harm to the developing skeleton (Figure 4, Figure 5, Figure 6 and Figure 7). Hip surveillance is accepted and implemented in all states in Australia and in many other countries with the aim of avoiding hip dislocation and the need for salvage surgery [11,13]. We propose similar surveillance of other joints, including the knee, ankle and foot, so that progressive MSP may be corrected with a simple surgery, predictable outcomes and a lower risk of surgical adverse events [1,3,16,17].

The limitations of the proposed classification are evident. MSP, like gross motor function, exists on a continuum, not as discrete groups with no overlap by age or clinical features [4,5,23]. The reliability we report may result from shared experiences in the MAL and might not be replicated in other centres or in other clinical groups. Future work will include refinement of the MSP staging. Validity will be examined in both cross-sectional and longitudinal studies of large groups of children attending the MAL.

Population-based studies have shown that changes in management in early childhood can prevent, or reduce to very low levels, examples of Stage 4 MSP; severe crouch gait and dislocation of the hip [24,25]. It is now time to see if other facets of Stage 4 decompensated MSP can be prevented in children with CP, to reduce long term pain and musculoskeletal morbidity in adult life [26].

## 5. Conclusions

Grading musculoskeletal pathology accurately might have a significant impact on the selection of appropriate management, the avoidance of iatrogenic harm and increased opportunity to optimise musculoskeletal health and to function in teenagers and young adults with cerebral palsy. For example, prior to the introduction and availability of botulinum toxin for the management of spastic equinus, in younger children with diplegia (Stage 1 MSP, Figure 2), many children in our centre had early lengthening of the Achilles tendons for equinus gait. This well-intentioned intervention was effective in correcting equinus in the short-term, but in the longer-term resulted in severe crouch gait in more than 40% of children with spastic diplegia [11,14]. Recognition of Stage 1 MSP, management by injections of botulinum toxin, can avoid this iatrogenic harm, which is illustrated in Figure 2. The counterpoint to this is that the popularity of botulinum toxin has resulted in the pendulum swinging in the opposite direction in that some children with fixed muscle tendon contractures and joint contractures, (Stage 2 and Stage 3 MSP) continue to receive injections after spasticity has transitioned to fixed contracture and at a time when injections are not beneficial, and orthopaedic surgery is required. (Figure 4 and Figure 5) It is our hope that the introduction of a staging system for MSP in children with cerebral palsy might lead to more appropriate management at each age and at each stage of MSP for the child with cerebral palsy, from birth to skeletal maturity.

## Figures and Tables

**Table 1:** Musculoskeletal pathology in children with spastic CP, according to anatomical level.

Level	Stage 1 Hypertonia Birth to Age 4 to 6 Years	Stage 2 Contractures Age 4 to 12 Years	Stage 3 Bony Deformity Age 4 to 12 Years	Stage 4 Decompensation Age 10 Years to Adulthood
Hip	Flexion/adduction, posturing. Clinically: scissoring.	Flexion/adduction contractures.	Increased FNA (>25°, hip IR > 2SDs internal 3DGA). Increased MP. Acetabular dysplasia.	Femoral head deformity. Acetabular deformity. Loss of articular cartilage. Arthrosis.
Knee	Spastic knee flexion. Hamstring spasticity. Full knee extension and occasionally recurvatum.	Hamstring contracture. Increased popliteal angle. Full knee extension or knee FDD <10°.	Knee joint contracture. Knee FFD: <20°. Mal-alignment: FNA + ETT. Genu valgum, genu varum.	Patella alta. Knee FFD > 20°. Patella fracture/avulsion. Arthrosis.
Ankle	Dynamic equinus. Ankle corrects to DF > 0° with knee extended.	Fixed equinus. Ankle dorsiflexion <0° with knee extended. If in doubt EUA is helpful.	Tibial torsion: External tibial torsion (ETT) > 20°. Internal tibial torsion (ITT) < 10° external.	Gross calcaneus, over-lengthened heel-cord. Deformity of talus. Arthrosis. LLD > 2.0 cms after skeletal maturity.
Foot	Flexible varus or valgus postures.	Partially fixed/flexible varus with muscle imbalance and/or contracture.	Fixed/stiff equino-varus, equinocavovarus. Pes valgus with LAD. Confirmed on radiographs and pedobarography.	Skin callosities and skin breakdown. Stress fractures, metatarsals. Deformed tarsal bones. Arthrosis.
Management	Tone management: Oral medications. Botulinum Toxin A (BoNT-A). Selective Dorsal Rhizotomy. Intrathecal Baclofen. AFOs and Physiotherapy.	Contracture surgery: Soft tissue surgery. Muscle recession. Tendon lengthening. Tendon transfers. AFOs and Physiotherapy.	Bony surgery: Osteotomies & stabilize joints. Usually includes soft tissue surgery: SEMLS/MLS. Guided growth FFD and LLD. AFOs and Physiotherapy.	Salvage surgery: Complex reconstruction (DFEO, PTS, PAO). Arthrodesis and arthroplasty. Assistive devices, wheeled mobility. Modify environment. Physiotherapy, Occupational Therapy.

Legend: FNA, femoral neck anteversion; MP, migration percentage; FFD, fixed flexion deformity; ETT, external tibial torsion; GSL, gastrocsoleus lengthening; BoNT-A, botulinum neurotoxin A; AFOs, ankle foot orthosis; SDR, selective dorsal rhizotomy; ITB, intrathecal baclofen; SEMLS, single event multilevel surgery; DFEO, distal femoral extension osteotomy; PTS, patellar tendon shortening; LLD, leg length discrepancy; LAD, lever arm deformity.

**Table 2:** Summary of patient data, for the 16 patients in the reliability study.

Pt No	Age in Years	Sex	TD	GMFCS	SGP	Spasticity	Contractures	Torsion	Decomp.	MSP	Rx
1	5 + 2	F	R Hemi	II	TE	R. GS, TP	R GS	None	None	2	BoNT-A
2	10 + 4	M	Diplegia	I	Jump	Bil GS, HS	Bil GS	Bil FNA	None	3	SEMLS
3	14 + 9	M	L Hemi	II	AE	L GS	3 levels	L FNA	L Hip OA	4	Salvage
4	1 + 8	F	L Hemi	I	TE	L GS	None	None	None	1	BoNT-A
5	7 + 4	F	Diplegia	II	AE	Bil GS, HS	GS/HS	None	None	2	SEMLS
6	1 + 10	F	Diplegia	I	TE	Bil GS	None	None	None	1	BoNT-A
7	12 + 3	M	Diplegia	III	Crouch	Bil HS	3 levels	FNA	Patellar #	4	Salvage
8	9 + 1	M	Diplegia	II	TE	Bil TP	Bil GS	Bil FNA	None	3	SEMLS
9	3 + 2	M	Quad	III	TE	3 levels	None	None	None	1	BoNT-A
10	8 + 3	F	Diplegia	II	Jump	GS	3 levels	None	None	3	SEMLS
11	7 + 6	F	R Hemi	II	TE	GS	GS, TP	None	None	2	SEMLS
12	11 + 9	M	Diplegia	II	Crouch	GS	3 levels	FNA, ETT	Knee FFD	4	Salvage
13	7 + 11	F	Diplegia	III	Jump	GS	3 levels	FNA	None	3	SEMLS
14	15 + 9	M	Diplegia	III	Crouch	GS, HS	3 levels	FNA	Knee FFD	4	Salvage
15	12 + 8	M	Diplegia	II	Crouch	HS	3 levels	None	Patellar #	4	Salvage
16	7 + 1	M	Diplegia	II	TE	GS	3 levels	Bil FNA	None	3	SEMLS

Abbreviations: TD, topographical distribution; Hemiplegia, diplegia, quadriplegia; GMFCS: gross motor function classification system; SGP, sagittal gait pattern; TE, true equinus; AE, apparent equinus; FNA, femoral neck anteversion; ETT, external tibial torsion; R, right, L, left; Bil, bilateral; GS, gastrocsoleus; HS, hamstrings; TP, tibialis posterior; OA, osteoarthritis; FFD, fixed flexion deformity. “Three (3) levels” refers to hip, knee and ankle/foot. Decomp., decompensation. Patellar #: Patellar fracture. BoNT-A, botulinum neurotoxin A; SEMLS, single event. Multilevel surgery can be soft tissue only (for Grade 2 MSP) or soft tissue and bony (for Grade 3 MSP); Salvage, salvage surgery. NB: Where treatment is described as SEMLS or salvage surgery, this does not preclude concomitant spasticity management.

## Data Availability

The data presented in this study are available on request from the corresponding author. The data are not publicly available due to patient privacy requirements related to the clinical data and videos used in the reliability study.
